# Deconstructing the Thymic Microenvironment Through Genesis to Senescence

**DOI:** 10.1111/imr.70048

**Published:** 2025-06-25

**Authors:** Michael D'Andrea, Kelin Zhao, Daniel H. D. Gray

**Affiliations:** ^1^ The Walter and Eliza Hall Institute Parkville Victoria Australia; ^2^ Department of Medical Biology The University of Melbourne Melbourne Victoria Australia

**Keywords:** epithelium, single‐cell technologies, stromal cells, T‐cell differentiation, thymus, tolerance

## Abstract

The thymus is essential for adaptive immunity, orchestrating the differentiation of hematopoietic progenitors into various T‐cell lineages. Thymic epithelial cells (TECs) impart this unique function by mediating the major checkpoints in T‐cell differentiation while also imposing stringent tolerance processes required to prevent autoimmunity. Achieving these feats requires extensive TEC specialization and the formation of distinct thymic microenvironments. These features change extensively throughout life, from the growth phases of the embryonic and perinatal thymus, into the steady‐state adult, through responses to acute injury and regeneration and, finally, during age‐related thymic involution. Here we review how hypothesis and technology have shaped the field's understanding of the thymic microenvironment. We focus on how the development of single‐cell technologies has revealed a remarkably diverse cellular landscape shaped by progenitor cell differentiation, TEC proliferation, AIRE‐mediated transcriptional processes, and the differentiation of thymic mimetic cell lineages.

## 1 Preamble

Jacques Miller's demonstration that the thymus was required for graft rejection, resistance to infection, the production of antibodies, and induction of immunological tolerance [1, 2, 3] established a key pillar of modern immunology [4]. The scientific and clinical ramifications of these foundational discoveries continue to shape vaccine design, transplantation, cellular therapies, and the treatment of autoimmune disease, cancer, and immunodeficiency. The decades since have revealed some of the remarkable mechanisms by which the thymic microenvironment orchestrates the differentiation of hematopoietic progenitors into multiple T‐cell lineages and the selection of a functional, diverse, and tolerant TCR repertoire.

Unlike other lymphoid tissues, the thymus is an epithelial organ. Unlike other epithelial cells, thymic epithelial cells (TEC) do not rest on a basement membrane; instead, they form a three‐dimensional network that facilitates interactions with thymocytes. These interactions are critical to virtually every important step in conventional and unconventional T‐cell differentiation (for an excellent introduction on this process, see ref. [5]). These TEC‐mediated steps include the chemoattraction and entry of hematopoietic progenitor cells from the blood; signals driving commitment to the T‐cell lineage and early CD3^−^CD4^−^CD8^−^ triple negative (TN) thymocyte differentiation; cytokines supporting thymocyte survival and proliferation; the presentation of unique peptide:MHC molecules to screen CD4^+^CD8^+^ double positive (DP) thymocytes auditioning their newly formed TCR complexes for positive selection; provision of ligands and growth factors supporting FOXP3^+^ regulatory T‐cell differentiation and expansion in the medulla; and the expression of a remarkably broad representation of peripheral tissue genes to directly and indirectly mediate negative selection processes in maturing CD4^+^ and CD8^+^ single positive (SP) thymocytes. Other thymic stromal elements, including fibroblasts, dendritic cells, macrophages, and endothelium also provide key signals and microenvironmental cues.

This review focuses on how non‐hematopoietic stromal cells constitute the unique structural and functional properties of the thymus. It is arranged in a loosely chronological order and highlights how advances in single‐cell technology, but also hypotheses and debate, have revealed the wonderful and sometimes weird ways that the thymic stroma brings about T‐cell differentiation and immunological tolerance.

## 2 In the Beginning…

The thymus has an epithelial origin. At embryonic day 9.5 (E9.5) in mice, a common parathyroid and thymic primordium is derived from out‐pocketing of the endodermal layer of the third pharyngeal pouch [6]. Development of this common primordium into the parathyroid or thymus is mediated by the early expression of two transcription factors: Glial cell missing homolog 2 (Gcm2) and Forkhead Box N1 (Foxn1), respectively [6]. Gcm2 is expressed in the antero‐dorsal area of the common primordium, whereas Foxn1 is confined to the ventral domain and drives thymic epithelial cell (TEC) differentiation [6]. The expression of these 2 transcription factors is also temporally separate, with upregulation of Gcm2 at E9.5 and Foxn1 48 h later [6]. Although Gcm2 and Foxn1 are necessary for the survival and differentiation of cells composing the parathyroid glands and thymus, neither has been implicated in the initial organ primordium fate [7, 8]. Studies of nude mice (*nu*/*nu*) with a congenital lack of *Foxn1* revealed that the absence of Foxn1 was responsible for athymia and for the characteristic hairless phenotype [9, 10]; however, the development of the thymic anlage was not impaired, suggesting that other factors upstream of Foxn1 may be responsible for organ fate [7, 11].

From E11.5, the common thymus‐parathyroid primordia becomes ensheathed by neural crest cell (NCC)‐derived mesenchyme and detaches from the pharynx in a process that involves apoptosis of the nearby ectoderm [12]. Several factors mediate this apoptosis. Deletion of *Hoxa3*, *Pax9*, and FGF pathway‐associated docking protein (*Frs2α*) from NC‐derived mesenchyme prevented the complete detachment of the thymic and parathyroid primordium from the pharynx [12]. Following this separation, the parathyroid glands remain in the posterior aspect of the thyroid where they exert hormonal control of calcium homeostasis. By contrast, the thymus migrates caudally and medially, reaching the superior mediastinum close to the pericardium [9, 13, 14]. Interestingly, it appears that this descent can lead to the blebbing off of cervical thymic lobules, which have been reported in mice and humans [15, 16]. In cohorts of pediatric patients referred for neck ultrasonography, the incidence of cervical thymic tissue “rests” was as high as 45%–60% [16, 17]. Although apparently benign, the mechanisms underlying the formation of cervical thymic tissue are not well understood. Factors such as Hoxa3 and BMP4 play roles in orchestrating thymic migration [7], in addition to NCC‐derived mesenchyme. Notably, deletion of ephrin B2 (a cell membrane protein involved in migration and adhesion) in NCCs impaired thymus migration with consequent formation of ectopic thymi [7, 18, 19].

Once the thymic rudiment is formed but before its descent is completed, lymphoid progenitor cells (LPC) begin migrating into the organ around E11.5 [12]. At this stage, the vasculature is not yet developed. LPCs expressing CCR7 and CCR9 enter through the dense mesenchyme under the influence of their ligands, CCL21 and CCL25, which are produced by the thymus and parathyroid rudiments [9, 12, 20]. This first wave of colonization is crucial for subsequent thymic organ patterning. Reciprocal interactions between thymocytes and TEC, termed thymic crosstalk, lead to the differentiation of medullary thymic epithelial cells (mTEC) and to cortical thymic epithelial cells (cTEC) [9, 21]. Several LPC ligands and receptors have been implicated in thymic crosstalk, among which members of the Tumor necrosis factor superfamily (TNFSF) are prominent. In particular, the differentiation of mTECs is mediated by the interaction between RANK‐RANKL, driven by lymphoid tissue inducer cells (LTIs) [22]. The differentiation of mTEC and cTEC marks the formation of the outer cortical and inner medullary regions necessary for distinct phases of T‐cell differentiation [9]. After E13.5, as the thymus attains its three‐dimensional structure and increases in volume, TEC progressively specialize to support the multistep process of thymocyte differentiation [9, 23].

## 3 Formation of the Thymic Microenvironment—Adult Contents

The fully developed thymus in adult mice and humans is composed of several different regions that orchestrate the discrete stages of thymocyte differentiation. TEC with distinct morphological characteristics were noted in early electron microscopy and immunohistological surveys of the thymus, detailing a variety of thymic “reticular cells” (e.g., [24, 25, 26, 27, 28, 29]). The creation and characterization of monoclonal antibodies against thymic stromal elements complemented the descriptions of morphological heterogeneity with defined molecular determinants. Particularly useful for distinguishing various TEC subsets were the ER‐TR and MTS series, the fucose‐binding lectin, UEA‐1, and antibodies detecting keratin subunits, MHC molecules, EpCAM, and cell surface proteins restricted to cTECs, such as CDR‐1 [30, 31, 32, 33, 34, 35, 36, 37, 38]. In addition, many non‐epithelial stromal elements were defined by related antibodies including thymic fibroblasts, identified by ER‐TR7 (recognizing type VI collagen) [39, 40] and MTS‐15 (recognizing the glycolipid Forssman antigen) [41] enabling dissection of their unique expression profile of chemokines, growth factors, and receptors critical for thymocytes and TEC alike. Studies with these reagents presaged an emerging appreciation of the multiple roles that fibroblasts play in thymic function [42]. Various syntheses that categorized the distribution of these thymic stromal cell reactivities (e.g., [37, 38]) coalesced into a highly influential diagram of the thymic microenvironment [43] that was quickly adopted by textbooks and continues to guide the field.

These insights revealed multiple distinct thymic microenvironments [44]. The cortico‐medullary junction (CMJ) serves as the primary entry point for T‐cell progenitor cells and is rich in blood vessels and adhesion molecules that facilitate cell recruitment [44]. The cortex can be divided into three main areas: the inner cortex, outer cortex and subcapsular cortex. Each region is responsible for different stages of T‐cell differentiation, including T‐cell lineage specification, T‐cell receptor β (TCRβ) and ⍺ (TCR⍺) recombination, positive selection, and CD4/CD8 lineage divergence [44]. In contrast, the medulla, comprising both outer and inner regions, is more loosely organized yet plays a key role in maintaining thymic architecture and regulating immune tolerance through crosstalk with developing thymocytes and negative selection, respectively [44].

A series of discoveries revealed the importance of thymic crosstalk for the formation of these mature microenvironments. Mice with defined blocks in T‐cell differentiation were found to also be deficient in the TEC populations that normally support them [45, 46]. For example, SCID mice incapable of generating TCRs (thereby blocking thymocyte differentiation at the CD3−CD4−CD8− (triple negative (TN) stage)) lacked fully formed thymic medullary regions [47]. Reconstitution of such mice with normal hematopoietic progenitors to restore thymocyte differentiation would, in turn, rescue TEC maturation and the adult thymic microenvironment [46, 48, 49, 50]. In addition to DP and mature SP thymocytes, other key mediators of thymic crosstalk included lymphoid tissue inducer (LTi) cells and unconventional T‐cell lineages, such as invariant NKT cells, that can also provide factors driving mTEC differentiation at various stages [51, 52]. These findings demonstrated a dynamism between the networks of cells composing the various thymic microenvironments and provided important clues into the molecular cues that control TEC differentiation (covered in more detail in Section 6).

**FIGURE 1:**
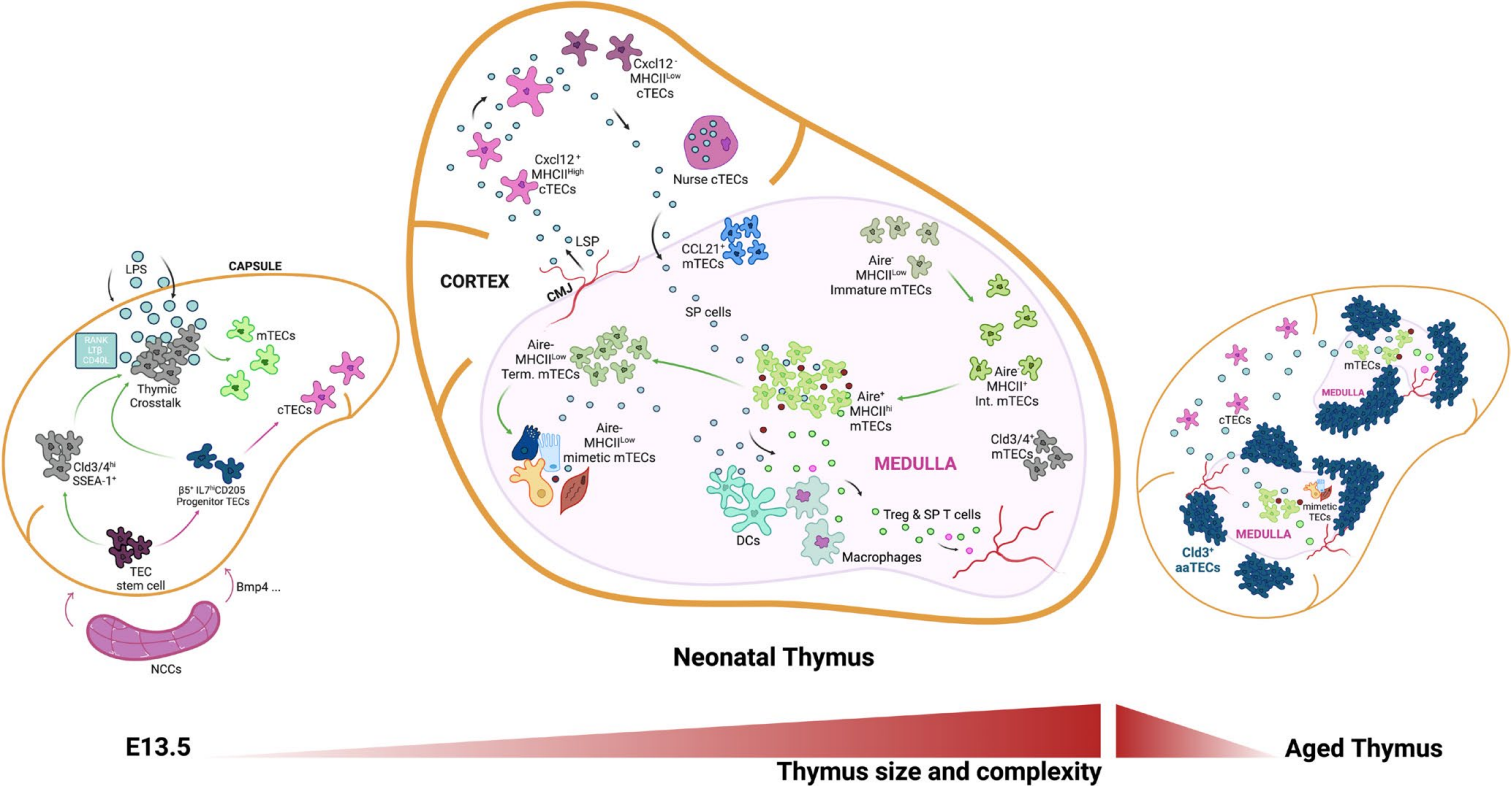
The thymic microenvironment during embryogenesis, neonatal and aged stages. The thymic microenvironment undergoes major changes through development, followed by a gradual decline during involution. aaTECs, age‐associated thymic epithelial cells; Cld3/4, claudin 3/4; CMJ, cortico‐medullary junction; DCs, dendritic cells; int. mTECs, intermediate mTECs; LSP, lymphoid seeding progenitors; NCCs, neural crest cells; SP, single positive T cells; term. mTECs, terminally differentiated mTECs.

Together, these discoveries laid the foundation for understanding the structural and functional complexity of the thymic microenvironment (Figure 1). However, major technical challenges held the field back. In contrast to the field of lymphocyte biology, it was very difficult to isolate and purify thymic stromal cells. Consequently, there were few quantitative studies of TEC dynamics and no capacity to isolate rare subsets for molecular or functional analysis. Moreover, there were no culture systems that could support adult thymic stromal cell function, nor was there any equivalent to adoptive transfer systems that could interrogate the function of purified subsets in vivo. Innovative new methods were required to overcome or bypass these limitations and enable reductionist approaches to better understand thymic function.

## 4 Dismantling the Thymic Microenvironment

### 4.1 Isolating Thymic Stromal Cells

The discovery that thymic microenvironments relied upon crosstalk with distinct thymocyte subsets hinted at a degree of dynamism in stromal cell populations. However, the histological approaches employed at the time were not well suited to resolving population dynamics or the molecular basis of thymic stromal cell function. Flow cytometry offered the prospect of leveraging the growing compendium of antibody reagents distinguishing thymic stromal cells to quantify and purify these populations. However, in contrast to the ease of obtaining lymphocyte suspensions that propelled other domains of immunology and hematology research, stromal cells presented a challenge due to their relative scarcity, complex morphology, and tight intercellular associations.

An essential technical innovation was the isolation of single‐cell suspensions of viable stromal cells that were amenable to labeling with fluorescent conjugates and flow cytometry. An early study by Wekerle et al. [53] developed a protocol combining trituration of thymic fragments from adult mice with trypsin digestion to enrich for thymic reticular epithelial cells. This protocol enabled analysis of stromal cell suspensions by immunohistochemistry, flow cytometry, light and electron microscopy, leading to the discovery of thymic nurse cells [54, 55]; large cortical epithelial cells that internalize up to 50 thymocytes within specialized cytoplasmic vacuoles [56, 57, 58, 59].

Refinements to this protocol, including the use of “gentler” digestion enzymes that break down the extracellular matrix (collagenase or a mixture of collagenase and dispase), revealed a broader range of thymocyte/stromal cell interactions, including multicellular complexes termed 'rosettes' composed of thymocytes interacting with macrophages, epithelial, or dendritic cells [60]. Although closely associated, the thymocytes from these rosettes remained outside of the stromal cells and were therefore distinct from thymic nurse cells. Rosettes could be disrupted by trypsin or by the calcium chelator, EDTA, suggesting that integrin‐mediated adhesion was important for their formation and preservation [60, 61]. Andrews and Shortman developed an ingenious technical innovation termed zonal unit‐gravity elutriation that could substantially enrich thymic nurse cells and rosettes from adult thymus suspensions on the basis of their density [62]. This technique, among other innovations, enabled higher resolution analysis of the thymocytes associated with rosettes. These experiments demonstrated that, although the interacting thymocytes were likely in close contact upon isolation, they did not reflect any particular stage of differentiation [60, 61]. Yet, analysis of rosettes from hematopoietic chimeras made with congenic mice enabled a kinetic analysis to infer the timing of lymphostromal interactions [63], providing insights that were consistent with thymic crosstalk.

Despite the capacity to isolate and enrich for thymic stromal cells, it was not possible to recapitulate all the stages of thymocyte differentiation in vitro [64, 65]. This impasse was due to a number of factors, including difficulties in maintaining the viability of thymic stromal cells and the loss of *Foxn1* and MHC expression on TEC grown as monolayers [66]. The inability to test the capacity of purified stromal cell populations to support key stages of thymocyte differentiation left open numerous critical questions about the cellular mediators of thymic function. A remarkable innovation that overcame some of these challenges was the development of an in vitro system that supported the growth and differentiation of defined populations thymic stromal cells. Intact thymi from embryonic mice were cultured with deoxyguanosine to deplete most thymocytes, then trypsin digests were subjected to immunomagnetic depletion of residual bone marrow‐derived cells to enable purification of defined stromal cell populations. The embryonic stromal cells could be reaggregated with thymocytes and maintained in similar conditions to fetal thymic organ culture [67], forming 3D structures that recapitulated the early thymic microenvironment and supported thymocyte differentiation and positive selection [68]. This system enabled the demonstration that both fibroblasts and MHC II^+^ TEC were required for early TN thymocyte differentiation, whereas MHCII^+^ TEC alone were sufficient to mediate thymocyte positive selection [69]. Reaggregate thymic organs cultures (RTOC) [70] remain an important reductionist tool for understanding TEC differentiation and function [71].

### 4.2 Parsing Out Epithelial Cell Heterogeneity

The next key development in the single‐cell analysis of thymic stromal cells was the application of the array of immunoconjugates developed to characterize the thymic microenvironment to elucidate TEC heterogeneity using flow cytometry. These were derived from surface reactive monoclonal antibodies from the ER‐TR and MTS series mentioned above [35, 36] and numerous others characterized, for example, [31, 72, 73]. Early efforts to dissect TEC heterogeneity in the adult thymus were stymied somewhat by high levels of autofluorescence and shared expression of antigens by thymic stromal cells and haematopoietically‐derived cells [74]. Multiparameter flow panels including anti‐CD45 conjugates bypassed these issues and enabled distinction of major cTEC and mTEC populations [75, 76, 77].

Yet, these protocols were technically challenging which restrained the broad uptake of flow cytometric analysis of thymic stromal cells. The use of different isolation strategies, a variety of enzymes, and the lack of a standard phenotypic schema contributed to highly variable outcomes. Again, inspired by studies of dendritic cells [78], systematic analyses of digestion isolation processes, epitope sensitivities, enrichment and purification protocols, stromal cell phenotype and quantification of TEC from adult and neonatal mice helped to standardize approaches in the field [77, 79]. These studies also revealed heterogeneity among cTEC and mTEC in their cell surface expression of key mediators of thymocyte selection, such as MHC II and costimulatory molecules (previously demonstrated by immunohistology [80, 81]), which would come to denote key functional and differentiation markers. These innovations were followed by numerous refinements that have improved the speed of isolation, cell yields, and viability [82, 83, 84, 85, 86, 87], making flow cytometric analysis and purification of thymic stromal cells a standard analytical tool in the field. The capacity to resolve TEC at the single‐cell level and purify them has led to major breakthroughs in our understanding of TEC differentiation, function, and immunological tolerance.

Yet, variable cell recoveries and under‐representation of stromal cells remain key limitations with these approaches. Several studies using genetic reporters and imaging approaches have quantified over 2 million TEC per adult mouse thymus [88, 89, 90, 91]; roughly an order of magnitude greater than estimates derived from enzymatic digestion and flow cytometry. Cortical TEC are particularly under‐represented by these protocols, likely due to their large size, more complex morphology, and associations with thymocytes. For example, most of the thymic nurse cells mentioned above are far too large for resolution by conventional flow cytometry. A challenge for the future is to develop new approaches to enable single‐cell isolation of thymic stromal cells that provide better recovery and improved viability, particularly of rare populations.

## 5 Promiscuous Gene Expression and AIRE

Although the thymus was recognized as playing an important role in inducing immunological tolerance during thymocyte differentiation, an outstanding question remained: how was tolerance to tissue‐specific or developmentally regulated proteins induced? The prevailing view was that the thymus, as a lymphoid organ, was unable to impose central tolerance to such antigens; therefore, peripheral tolerance mechanisms must be responsible. However, various reports emerged demonstrating that mice expressing transgenes under the control of tissue‐specific promoters also exhibited expression in the thymus, which could be tolerogenic (reviewed by [92]). The question of whether this expression might simply represent a transgenic artifact was settled when the transcription of endogenous *Ins2* (encoding insulin) and other pancreatic genes was detected in rare thymic cells in mice and rats [93, 94, 95]. Moreover, cells transcribing insulin were also detected in the thymus of humans, with the amount of expression linked to the risk of developing type I diabetes [96, 97]. A key step forward was the precise identification of these thymic cells by Derbinski et al., who performed PCR analysis of FACS‐purified thymic stromal cells, including cTECs and mTECs, revealing that insulin expression was confined to mTECs [75]. More remarkably, they conducted a broad survey of other proteins expressed in the pancreas, liver, thyroid, central nervous system, and retina; all were expressed either predominantly or exclusively by mTECs. These findings demonstrated the phenomenon of promiscuous gene expression (PGE), the broad expression of tissue‐specific genes by mTECs [98]. Subsequent studies tallied the transcription of thousands of tissue‐specific genes in mTECs; up to 90% of the protein‐coding genome [99]. The discovery of PGE had potentially significant implications for our understanding of immunological tolerance [100].

A notable control for the purity for stromal cell purification strategy in the Derbinksi et al. paper was transcription of *Aire* [75]. Mutations in this gene were discovered as the cause of the monogenic syndrome, autoimmune polyglandular syndrome type 1 (APS1) (also known as autoimmune polyendocrinopathy‐candidasis‐ectodermal dystrophy, or APECED) [101, 102], an autosomal‐recessive disorder featuring multi‐organ autoimmunity. An important clue into how this transcription factor might be linked to immunological tolerance was that its expression was predominantly restricted to mTECs [103, 104]. The discovery of broad PGE in the same cell population led to speculation that AIRE might be involved in this process [75]. Almost a year later, this link was confirmed. Anderson et al. created *Aire*‐deficient mice and demonstrated that they succumbed to multi‐organ autoimmunity [76, 105]. Importantly, transplantation of thymic stroma from *Aire*‐deficient mice into athymic recipients was sufficient to induce autoimmunity, localizing AIRE's tolerogenic activity to mTECs. FACS purification of mTECs for microarray analysis revealed that PGE was substantially diminished (but not completely lost) in *Aire*‐deficient mice, demonstrating an essential role for AIRE in tissue‐specific gene expression by mTECs and central tolerance [76]. These discoveries changed the field by demonstrating that the thymus provides a preview of the peripheral self that is essential for the prevention of autoimmunity.

In terms of cellular mechanism, subsequent studies demonstrated that the expression of AIRE‐regulated model antigens by mTECs could induce thymocyte deletion [106, 107, 108] or the selection of FOXP3^+^ regulatory T cells [109, 110, 111]. The severity of autoimmune phenotypes in *Aire* ^−/−^ mice varied based on the genetic background, largely due to MHC [112]. For instance, the phenotype in C57Bl/6.*Aire* ^−/−^ mice was mild (although substantial, including retinopathy and infertility) relative to NOD. *Aire* ^−/−^ mice, which succumbed to fatal exocrine pancreatitis, among other severe impacts [112]. The production of IFNγ is the key effector mechanism mediating disease in murine models and humans, with ruxolitinib‐induced JAK–STAT blockade demonstrating promising efficacy in ameliorating disease [113]. Surprisingly, and in contrast to most autoimmune diseases and models, there appears to be no requirement for innate immune signaling or the microbiome in the manifestation of autoimmunity in *Aire* ^−/−^ mice [114], emphasizing the importance of AIRE‐mediated central tolerance in preventing autoimmunity. Nevertheless, peripheral tolerance mechanisms provide a potent buttress against fatal autoimmunity in *Aire* ^−/−^ mice, with signaling through PD‐1 or CBL‐B restraining the activation of CD4^+^ T cells [115, 116, 117]. Overall, these findings provide insight into the processes and pathways most sensitive to dysregulation in the context of sub‐clinical defects in AIRE‐mediated tolerance.

How does AIRE mediate such broad PGE in TEC? AIRE's structure and nuclear localization suggested that it acts as a transcription factor. However, whether AIRE directly mediates PGE or somehow influences TEC differentiation to enable other mechanisms to elicit PGE was initially unclear. Two hypotheses emerged. The progressive restriction model was founded on observations of TEC with morphology that distinctly resembled cells from diverse peripheral tissues; for example, ciliated cells, myoid cells, epithelia with villi (e.g., [25, 28, 29]). Farr and colleagues proposed that a TEC progenitor cell with high potential could give rise to diverse lineages of epithelia that became progressively restricted to a given peripheral cell fate [118, 119]. These differentiated TEC composed a mosaic that collectively presented the peripheral proteome to thymocytes. AIRE was proposed to be expressed by TEC progenitors and cause or influence the adoption of alternative cell fate [120]. In this progressive restriction model, PGE would be expected to reflect concerted differentiation programs at the single‐cell level. Consistent with this hypothesis, TEC compositional changes were observed in *Aire*‐deficient mice [121, 122].

An alternative hypothesis was that PGE was the consequence of a stochastic process of transcriptional de‐repression that accrued as mTEC matured [120, 123]. AIRE would act in terminally differentiated mTEC to greatly amplify PGE such that individual cells would end up producing a diverse panoply of transcripts, rather than a concerted program reflecting other cell lineages. These competing views provided a very useful hypothetical framework for determining how PGE operates in TEC and highlighted large gaps in our understanding of TEC differentiation. In the two decades since these papers [120, 123], innovative mouse models, culture systems, and single‐cell technologies have shed new light on TEC differentiation and PGE to arrive at an amazing new consensus: that aspects of both models are correct.

## 6 Thymic Epithelial Cell Differentiation—Where Does AIRE Fit?

Recent comprehensive reviews on this topic are available [105, 124, 125], so here we focus on how flow cytometric and single‐cell transcriptomic analyses of TEC have provided new perspectives on their heterogeneity that have informed novel schemas of their differentiation hierarchy. Early phenotypic analysis revealed highly dynamic changes in TEC composition across thymic ontogeny, including a skewing toward higher numbers of mTEC over cTEC in the adult [126]. All EpCAM^+^ TEC expressed high cell surface levels of MHC II in early life (TEC^high^) followed by the emergence of CD80^−^ MHC II^low^ subpopulations (TEC^low^) in both mTEC and cTEC from young adult mice [123, 126]. High rates of proliferation were linked to this dynamic, with Ki67 staining and BrdU/EdU incorporation studies identifying the highest rates of proliferation within the mTEC^high^ population [126, 127, 128]. The notion that cTEC^low^, cTEC^high^, mTEC^low^ and mTEC^high^ might represent distinct stages of TEC differentiation was supported by the analysis of mice with defined blocks in thymocyte crosstalk signals (*Rag1* ^−/−^, *Tcra* ^−/−^, *Cd40lg* ^−/−^) or TEC differentiation (*Relb* ^−/−^) [126]. These data suggested a phenotypic progression from cTEC^high^ → cTEC^low^ → mTEC^high^ → mTEC^low^; however, whether these requirements reflected differentiation or survival, or whether other aspects of TEC phenotype within these populations were altered, was not clear at the time. Subsequent studies established the importance of RANKL from mature thymocytes in the expansion of the mTEC^high^ population via RANK signaling and activation of the non‐canonical NFκB pathway in TEC [22, 129, 130, 131].

AIRE was found to be expressed within the mTEC^high^ subset, coincident with the highest PGE [75, 76, 123]. These findings brought the relationship between mTEC^low^ and mTEC^high^ to the fore. The progressive restriction model predicted that mTEC^high^ would give rise to mTEC^low^ (mirroring the relationship implied by the ontogenic analysis [126]); the terminal differentiation model predicted the opposite. Several groups then found that AIRE‐expressing mTEC^high^ could be derived from AIRE^−^ precursors. Rossi et al. focused on the differentiation of the first AIRE‐expressing cells in the embryonic thymus using genetic models and RTOC systems, clearly demonstrating that CD80^−^AIRE^−^ mTEC matured into CD80^+^AIRE^+^ mTEC under the influence of LTi‐derived RANKL signals [22]. Subsequent studies of the steady‐state adult thymus employed in vivo BrdU pulse/chase experiments and a modified RTOC system to test the differentiation potential of mTEC^low^ from adult mice, concluding that AIRE^+^ mTEC^high^ were non‐dividing [132] (contrasting a highly proliferative, transit amplifying population of AIRE^−^mTEC^high^ cells; a finding that has been contested [127]) and could be derived from mTEC^low^ precursors [128, 132]. Other data also suggested that AIRE expression led to apoptosis of mTEC based on cellular turnover estimates and AIRE transduction experiments in cell lines [132, 133]. These features, combined with insights into the molecular mechanisms of AIRE‐mediated transcription suggesting a stochastic rather than ordered program of gene expression, favored the terminal differentiation model [134].

This view changed with insights gained from detailed temporal analyses and genetically modified mouse models that enabled tracking of the fate of *Aire*‐expressing cells [135, 136, 137, 138, 139]. These studies revealed a subpopulation of mTEC^low^ was derived from cells that had previously transcribed *Aire*. Further phenotypic correlates and transcriptional analyses of these differentiation steps have revealed that post‐AIRE mTEC exhibit features of terminal differentiation of keratinocytes (and other cell lineages), typified by involucrin and keratin‐10 expression [140, 141, 142], potentially destined to become Hassall's corpuscles. The demonstration that there was “life after AIRE” (for at least some cells) positioned AIRE as an intermediary, contradicting the terminal differentiation model (Figure 1).

Given this newfound diversity in the mTEC^low^ population, the question arose: what subpopulation of mTEC^low^ gives rise to AIRE^+^ mTEC^high^ cells? Embryonically derived mTEC progenitors are capable of giving rise to both AIRE^−^ mTEC^low^ cells that express the chemokine, CCL21 [71, 125, 143] (important for the migration of maturing thymocytes into the medulla [144]) and AIRE^+^ mTEC^high^. In the postnatal thymus, recent insights into the relationship between mTEC^low^ and mTEC^high^ using fate mapping, cell ablation, and scRNA‐seq analyses suggest that a proliferative, intermediary population may directly become CCL21^+^ mTEC^low^ or AIRE^+^ mTEC^high^ (which, in turn, can become post‐AIRE cells) [145, 146]. Such a model has attractive parallels with transit amplifying populations in other tissues. It may also be consistent with a prior finding that some mTEC^low^ from adult mice can directly give rise to AIRE^+^ mTEC^high^ in RTOC [132]. Short‐term BrdU/EdU labeling experiments have shown the highly proliferative, putative transit amplifying population of mTEC has intermediate amounts of surface MHC II [127, 132]; some of these cells would be included in an mTEC^low^ gating scheme. Future experiments taking advantage of more precise phenotypic distinction and fate mapping approaches will be required to further test this model of postnatal mTEC differentiation.

## 7 Life After AIRE and Thymic Mimetic Cells

These roadmaps of TEC differentiation set the scene for new single‐cell technologies that revealed an unprecedented level of heterogeneity among TEC, posing new questions about their function and population dynamics. Initial studies employed single‐cell RNA sequencing (scRNA‐seq) on thousands of purified thymic stromal cells, revealing transcriptional profiles that mapped relatively well to the known cTEC and mTEC subpopulations [145, 147, 148, 149, 150]. These studies also uncovered informative new combinations of surface proteins to further categorize mTECs. For example, Bornstein et al. delineated a flow cytometric staining schema for mTEC I‐IV based on the major transcriptional states they uncovered and the inferred differentiation progression [147]. An outlier was the mTEC IV population, which did not resemble any described TEC population but, remarkably, had more in common with intestinal tuft cells. A contemporaneous study used a fate mapping allele to sort purify mTEC^low^ cells 7 days after having expressed *Aire* and identified transcripts associated with cornified epithelia and tuft cells [151]. Both studies found that thymic tuft cells represented a distinct population of mTEC^low^ that had the morphology and transcriptional hallmarks of tuft cells from elsewhere in the body, including a reliance on the transcription factor *Pou2f3* for their differentiation [147, 151]. One of these hallmarks included the production of IL‐25 which was found to modulate thymic unconventional and innate‐like lymphocytes [147, 151], and mediate central tolerance to this cytokine [151].

These surprising discoveries were reminiscent of early findings of cells with morphologies characteristic of peripheral lineages; ciliated, villous, and myoid cells were all found in the medulla, in addition to the striking Hassall's corpuscle structures [24, 25, 26, 27, 28, 29]. These findings also refocused attention on the mTEC^low^ subset. Multi‐omic analysis of higher numbers of sort‐purified mTEC^low^ revealed the presence of the transcriptional analogues of a large variety of peripheral cell lineages beyond tuft or cornified lineages, including corneocytes, myocytes, and microfold (M‐cell) mTECs [152, 153]. These thymic mimics of numerous peripheral epithelial cell lineages have been termed “mimetic” cells [119]. Transcription factor motif enrichment analysis showed that the chromatin landscape of mimetic cells is shaped by, and relies upon, the same transcription factors as their peripheral counterparts [152, 154]. The majority of mimetic cells likely derive from post‐AIRE mTECs, as they were found to be enriched in the Pdpn^−^CD104^−^MHCII^low^ mTEC compartment [153] (Figure 1). This finding raised the question of whether AIRE is responsible for the differentiation of mimetic populations. Analysis of TEC from *Aire* ^*−/−*^ mice showed that, although some mimetic cell subsets were substantially reduced, other mimetic cell types remained, indicating that mimetic cells are not strictly dependent on AIRE [151, 153]. The persistence of these mimetic cells explains why peripheral tissue antigen (PTA; also termed tissue‐specific antigens, TSAs) expression was not completely absent from TEC from *Aire*‐deficient mice [151, 153].

These discoveries unveiled the molecular basis of a curious feature of the thymus; what about mimetic cell function? Thymocytes reactive to a transgenic reporter expressed in mimetic populations underwent central tolerance [152], extending on earlier evidence that thymic tuft cell‐derived IL‐25 could prevent elicitation of auto‐antibodies [151]. Mice lacking a specific type of endocrine mimetic TECs developed auto‐antibodies against endocrine tissues, although they did not exhibit overt lymphocytic infiltration [153]. However, evidence of functions beyond immunological tolerance have also been found. Thymic M‐cell mimetics induce IgA production from thymic B cells in a process analogous to that observed in the intestine [153]. In addition, a recent study found thymic tuft cells could promote thymic regeneration after dexamethasone‐induced thymic atrophy through the production of IL‐25 and activation of a type 2 immune response [155]. By contrast, in irradiation‐induced thymic atrophy, mimetic tuft cells appeared to be dispensable for thymic regeneration, with other cells (eosinophils, fibroblasts, and ILC2s) involved in this distinct damage response [156].

Lastly, a deep characterization of mimetic TECs from pediatric human tissue has revealed many homologues of the subpopulations discovered in mouse but with a much higher representation of neuroendocrine and myoid mimetic cells [157]. Although rare, some dysferlin‐positive mimetic TECs were associated with Tuft‐derived choline acetyltransferase CHAT enzyme and alpha bungarotoxin, which binds to the nicotinic acetylcholine receptor (AChR) [157]. This structure appears to resemble the neuromuscular junction, suggesting that mimetic populations can also recapitulate intercellular associations relevant to immunological tolerance; in this case, potentially the prevention of myasthenia gravis.

Taken together, mimetic mTECs represent a diverse and functionally distinct population within the thymus. Their ability to mirror peripheral cell characteristics and contribute to central tolerance highlights their importance in shaping adaptive immunity. Further research is required to fully understand their origins, regulatory mechanisms, and broader immunological significance.

## 8 Thymic Self‐Representation and Peripheral Tolerance

AIRE‐mediated gene expression, thymic mimetic cells, and other antigen presenting cells evoke a remarkably broad representation of peripheral self in the thymus [158]. These mechanisms provide the substrates for central tolerance. The relative impact of peripheral antigen expression on the various tolerogenic mechanisms in the thymus has been a key question in the field. Thymocytes with TCRs that have an inappropriately high avidity for self‐peptide:MHC complexes presented by thymic APCs are subject to multiple processes to defuse the risk of autoimmunity. These include apoptotic deletion [159], adoption of a FOXP3^+^ regulatory or unconventional T‐cell fate [160], anergy [161], or clonal “eviction” [162]. The most prominent mechanisms of central tolerance linked to AIRE‐regulated antigens have been deletion [106, 107, 163] and Treg induction [109, 111]. A recent study has shed light on how apparently analog TCR signals connect to some of these mechanisms via the nuclear orphan receptors, Nr4a1 (aka Nur77) and Nr4a3 (aka Nor1) [164]. Nr4a1 expression is highly sensitive to the avidity of TCR stimuli [165] and, with Nr4a3, was found to regulate expression of BIM [164] via a thymocyte‐specific enhancer element [166]. BIM is a pro‐apoptotic BH3‐only protein that initiates most thymocyte deletion via the intrinsic or BCL‐2‐regulated pathway of apoptosis [167, 168]. Other components of the Nr4a1/Nr4a3 transcriptional program included upregulation of regulators of a T‐cell anergy program, including Cbl‐B, PD‐1, and SOCS proteins [164], highlighting further connections to the mechanisms of negative selection. The extent to which central tolerance to peripheral antigens engages the various negative selection mechanisms remains an open question that more extensive analyses of TCR repertoire may shed light on.

More broadly, the unique mechanisms controlling thymic self‐representation have provided an opportunity to explore the interplay between central and peripheral mechanisms. Defects in thymic self‐representation can be fatal. As outlined above, mutations in *Aire* impair central tolerance and lead to autoimmunity. In humans, *AIRE* loss‐of‐function causes Autoimmune Polyendocrine Syndrome Type 1 (APS‐1) characterized by multi‐organ autoimmunity [101, 169]. In mice, *Aire* deficiency in the thymic stroma was sufficient to cause spontaneous, multi‐organ autoimmune disease [76], and this phenotype varied in severity based on the genetic background of *Aire* ^−/−^ animals [112]. The risk posed by T cells reactive to AIRE‐dependent antigens is apparently very high because, in contrast to most models of autoimmunity, peripheral triggers and innate immune conditioning are not necessary for autoimmune disease. *Aire* ^−/−^ mice raised in germ‐free conditions or challenged with innate immune stimuli or rendered deficient in *Myd88* or *Trif* developed autoimmune manifestations of the same severity as controls [114]. This lack of peripheral constraint of such T‐cell clones is likely most relevant to TCR specificities that cannot be deleted or otherwise tolerized due to complete loss of peripheral antigen expression in the thymus (e.g., IRBP‐reactive clones that drive the retinopathy in *Aire* ^−/−^ mice [163]). However, it is important to note that the expression of many peripheral genes by TECs is only partially reduced in the absence of AIRE [76, 99].

The engagement of other tolerogenic safeguards has been explored in *Aire* ^−^/^−^ mice on the C57BL/6 or B10/Br backgrounds, which exhibit milder autoimmune phenotypes relative to the fatal disease observed in mice on the NOD background [112, 170, 171]. Teh et al. [115] found that a regulator of T‐cell anergy, CBL‐B, was essential for protecting *Aire* ^−^/^−^.B10/Br mice from fatal exocrine pancreatitis, demonstrating a key axis of cooperation between central and peripheral tolerance. Similarly, we found that *Cblb* ^−/−^ *Aire* ^−/−^ mice on a C57BL/6 background had more severe infiltration of a variety of organs [116]. This concept was further extended by the finding that double knockout mice lacking both AIRE and PD‐1 (*Aire* ^−/−^ *Pdcd‐1* ^−/−^) developed severe systemic autoimmunity, including fatal exocrine pancreatitis [116]. These phenotypes contrasted the modest impact we observed with compound deletion of *Bcl2l11* (the gene encoding BIM) with *Aire*, *Cblb*, or *Pdcd1*. These findings underscore the importance of peripheral tolerance safeguards in restraining autoreactive T cells that escape central deletion. Notably, these findings mirror the immune‐related adverse events observed in patients treated with checkpoint inhibitors (e.g., anti–PD‐1 or anti–CTLA‐4), further emphasizing the functional interplay between central and peripheral tolerance mechanisms [172]. Contextualizing the role of mimetic cells in immunological tolerance and uncovering additional pathways that maintain immune homeostasis, especially under conditions of genetic predisposition or environmental stress will be interesting themes for future research.

## 9 Cortical Unknowns

In parallel with the field's newfound appreciation of mTEC heterogeneity, investigations of their cortical counterparts have revealed an apparent division of labor that raises still more questions about this essential microenvironment. Cortical TECs mediate multiple critical early steps in thymocyte differentiation, including the provision of: Notch ligands required for T‐cell lineage commitment; key growth factors (such as SCF and IL‐7); chemokine cues (CXCL12) and unique antigen processing functions to support positive selection of DP thymocytes [5]. Early indications that some level of cTEC heterogeneity exists came from immunohistochemical analysis of thymic sections using multiple monoclonal antibodies [43]. Subsequent single‐cell flow cytometry studies have elucidated molecular diversity among cTECs, many relevant to their functions in orchestrating early thymocyte differentiation, including: MHC II, VCAM‐1, Delta‐like ligand‐4, IL‐7, Ly51, DEC‐205, Sca‐1, and α6 integrin [77, 173, 174, 175, 176, 177, 178, 179, 180]. Transcriptional analyses across distinct cortical regions (i.e., the outer, central, and peri‐medullary cortex) reinforced these observations, revealing region‐specific expression patterns [181]. New insights into the timing and control of cTEC heterogeneity were provided by a recent study using a *Cxcl12* ^DsRed^ genetic reporter mouse [177]. The chemokine, CXCL12, is expressed by cTECs and ligates CXCR4, providing an important migration cue for early thymocyte differentiation [182]. Perinatal cTECs expressed uniformly high amounts of *Cxcl12* ^DsRed^, followed by the emergence of *Cxcl12* ^DsRed^ negative cTECs under the influence of TN differentiation into DP thymocytes [177]. This study also found *Cxcl12* ^DsRed^ negative cTECs had downregulated FOXN1, coinciding with reduced expression of key target genes supporting very early thymocyte differentiation (e.g., *Dll4*) and positive selection (e.g., *Psmb11*, encoding the thymoproteasome subunit, β5t) (Figure 1). These findings of a transition in cTEC heterogeneity linked to functions in positive selection were supported by another study employing CITEseq and functional analyses [183]. This interspersal of crosstalk‐induced CXCL12^−^ cTECs with a lower potential for positive selection might reflect a need to create “space” for counter‐migratory TN thymocytes. Or perhaps it is necessary to accommodate other cortical stromal cell types, such as dendritic cells. Or this heterogeneity may reflect some other specialized function of CXCL12^−^ cTECs that remains to be determined.

Consistent with this diversity among mouse cTEC, three distinct cortical subpopulations have been identified in the human thymus [184, 185]: cTEC I, cTEC II, and cTEC III. The cTEC I subset closely resembles the previously described perinatal cTEC population in mice and is characterized by a proliferative transcriptional signature [145]. cTEC III appear analogous to the mature cTEC population in mouse, with a localization at the subcapsular and outer cortical regions of pediatric (but not embryonic) thymus [184, 185]. The cTEC II population appears to constitute a developmental intermediate between cTEC I and cTEC III [185].

Thymic nurse cells represent another interesting element of cTEC heterogeneity. These cTEC enclose up to 50 thymocytes and were discovered as prominent features of stromal cell enriched enzymatic digests [56, 57, 58, 59]. A study employing a β5t reporter used optimized isolation protocols for flow cytometry to quantify that thymic nurse cells compose approximately 10% of cTEC [59]. This study also resolved several technical concerns regarding the nature of thymic nurse cells by resolving them in situ using confocal and intravital microscopy. Thymic nurse cells were found to arise upon DP thymocyte differentiation, with sustained interactions promoting their engulfment and secondary TCRα chain rearrangements [59]. Further investigation of these fascinating structures and cTEC heterogeneity more broadly will require specialized analytical approaches to better resolve these complex cells.

## 10 TEC Progenitors

The identity and role of TEC progenitors in thymic development and postnatal TEC dynamics have been extensively studied. The resolution of putative progenitor populations, particularly in the postnatal thymus, has been complicated by their relative rarity and difficulties in propagating TEC differentiation in vitro. RTOC experiments and in vivo fate mapping demonstrated that a common progenitor of cTEC and mTEC exists in the embryo and that such progenitors could be made to persist in the postnatal thymus [186, 187]. The search for ways to prospectively isolate embryonic TEC progenitors led investigations of the antibodies, MTS‐20 and MTS‐24. Both recognize Placenta‐expressing transcript, PLET‐1, a protein highly expressed by embryonic TEC which becomes progressively more restricted to isolated cells in the medulla in the adult [36, 188, 189]. Two studies found that RTOC derived from populations of MTS‐20/24^+^ TEC purified from E12 or E15 thymi could give rise to a complete thymic microenvironment upon grafting into mice [186, 190]. However, a subsequent report titrated the number of cells used to form these RTOC and found that MTS‐24^−^ from E15 thymus could also generate cortical and medullary TEC microenvironments when grafted in sufficient numbers [191]. It has since emerged that cTECs and mTECs arise from earlier bipotent progenitors that express the cTEC‐associated proteins β5t, IL‐7 and CD205 [192, 193, 194], leading to the concept of a cTEC‐like intermediary [125].

Nevertheless, whether a similarly bipotent pool of progenitors supports postnatal TEC differentiation remains an important question. Reactivation of *Foxn1* in single TEC, previously retained within the postnatal thymic microenvironment in a FOXN1^−^ state, gave rise to both mTECs and cTECs, demonstrating that such a population could contribute to steady‐state thymic lymphopoiesis [187]. Building on these observations, another study found evidence for an EpCAM^−^ Foxn1^−^ stem cell population among CD45^−^ stromal cells in the postnatal thymus capable of forming “thymospheres” using stem cell spheroid culture systems developed to study other epithelial tissues [195]. Cell populations derived from these cultures could give rise to cTECs and mTECs in vivo. A subsequent report found that thymospheres formed from these stromal populations were derived from mesenchymal stromal cells that could incorporate mature TEC into the structures, rather than the product of a FOXN1^−^ postnatal progenitor [196]. Therefore, the notion that retention of a FOXN1^−^ stem cell population in the postnatal thymus supports TEC differentiation remains an open question.

Using a different strategy, two studies identified cTECs subpopulations (albeit, with differing phenotypes) in the thymus of adult mice capable of giving rise to both cTECs and mTECs in RTOC and/or upon intrathymic injection [175, 197]. On the other hand, fate mapping studies of postnatal TEC that had expressed β5t (a unique characteristic of cTECs) did not find that substantial numbers of mTECs arose from such precursors [198, 199]. Using single‐cell RNAseq combined with in situ genetic barcoding, Nusser et al. identified distinct transcriptional signatures in potential TEC progenitors from the embryonic versus postnatal thymus [200]. Embryonic TEC progenitor‐like populations appeared more cTEC biased, whereas the postnatal counterparts appeared more mTEC biased [200]. The differences in the progenitor cell phenotypes identified in these various studies may reflect heterogeneity among cells capable of giving rise to cTECs and mTECs or variations in the read‐outs used and the location of the cells; that is, steady‐state turnover versus incorporation into RTOC mixtures or intrathymic injection.

TEC isolated from humans are more amenable to in vitro differentiation. CD49f^+^ TECs isolated from neonatal and pediatric human thymus possess clonogenic capacity in vitro and repopulation potential ex vivo. Notably, these clonogenic TECs display a transcriptional signature that aligns with com‐TECs (a population that shares transcriptional characteristics of both mTECs and cTECs) suggesting a potential stem or progenitor‐like capacity [184]. Within the cmTEC population, a subset expresses multiple keratin subunits, referred to as “polykeratin” cells. Single‐cell RNA‐seq analyses have also identified this population, finding transcriptional and phenotypic similarity with skin stem cells and a capacity to give rise to differentiated TEC [201]. Spatial transcriptomic analysis of the human pediatric thymus revealed the presence of mcTEC, an intermediate population, within niches in both the capsular and CMJ regions [185]. The capsular mcTECs displayed an apparent transcriptional bias toward cTEC differentiation, whereas CMJ mcTECs were either mTEC‐primed or remained uncommitted [185]. These findings further suggest that the thymic microenvironment determines whether progenitor cells have a bias toward differentiating into cTECs or mTECs.

Collectively, the data indicate that mature cTECs and mTECs derive from a common progenitor cell present in the embryonic thymus. In the adult thymus, the data suggest the bifurcation of cortical and medullary subsets is controlled by progenitors skewed toward a specific compartment. Precisely how postnatal TEC progenitors might contribute to the turnover of differentiated TEC during steady‐state, thymic involution or injury remain open questions. Recent scRNA‐seq analyses have revealed the emergence and expansion of progenitor‐like populations during aging [90, 145, 200]. Baran‐Gale et al. provided evidence that intertypical TECs act as progenitors of mature mTECs but progressively lose their differentiation capacity with age [145]. However, further studies using high diversity genetic barcoding and other strategies may provide a comprehensive map of TEC progenitor potential and actual contribution to thymic function in various states. Harnessing this understanding will likely open new strategies for reconstituting thymic function to overcome immunodeficiency.

## 11 Where Do They All Go? TEC Death

Measurements of TEC proliferation using Ki67 or BrdU incorporation in the adult mouse thymus revealed: (1) relatively high rates of cell division maintain their turnover at steady‐state, and; (2) the proportion of dividing TEC diminished during thymic involution [126, 128, 132]. These conclusions were supported by pulse‐chase experiments quantifying label‐retaining cells that were induced in vivo using a tetracycline‐inducible H2B‐GFP fusion protein [202]. This study also found evidence of higher rates of TEC turnover in female mice versus males and among mTECs versus cTECs [202]. Consistent with the latter finding, the majority of proliferating TEC detected shortly after a pulse of BrdU or EdU were MHCII^int^ mTEC [127, 132]. This high turnover of mTECs at steady‐state prompts the question of what happens to them all?

Possibilities include: (1) they undergo cell death and are engulfed by phagocytes, (2) they undergo a cornification process akin to skin keratinocytes, before being cleared, (3) they are ingested by thymic macrophages independent of cell death processes, mediated by “eat me” signals, (4) they emigrate from the thymus into the circulation. Most data on this topic concerns the first hypothesis. Several groups have assayed TEC isolated from adult mice with well‐established markers of apoptosis, such as phosphatidylserine (PS) exposure and activated caspase‐3 [140, 202, 203]; however, these data imply surprisingly high rates of apoptosis (up to 70% in a population of mTEC^low^ in one study). These findings raise the question of whether the enzymatic digestion processes necessary to isolate TEC for analysis may be inducing the apoptosis observed and/or PS flipping. Indeed, analysis of TEC apoptosis using TUNEL staining to detect DNA fragmentation (a feature of late‐stage apoptotic cells) in situ revealed low rates of TEC apoptosis (< 1%) [203, 204]. Although these studies focused on embryonic stages when little TEC apoptosis might be expected, they demonstrated a dynamic range for detection by comparison with genetic models that promoted cell death, such as p63 knockout mice. Senoo et al. also found the enrichment of phagocytes around dying TECs [204], consistent with heightened efferocytosis. This finding highlights a challenge with measuring cell death in situ, well‐known in thymocyte selection [205]; phagocytes are often better at detecting early apoptotic cells than the current tools we have. There is an opportunity to apply newly developed approaches for resolving various cell death modalities and phagocytosis to better understand TEC turnover.

Other studies have attempted to infer cell death control in TECs by employing genetic models that manipulate various survival pathways. An essential role for the pro‐survival BCL‐2 family member, MCL‐1, in TEC was revealed when TEC‐specific or inducible ablation was found to cause their death via the BAX/BAK‐mediated intrinsic pathway of apoptosis, leading to severe thymic atrophy [206]. Curiously, the pro‐survival proteins, BCL‐2 and BCL‐xL, were found to be dispensable for steady‐state TEC survival. MCL‐1 expression in TEC could be induced by EGF‐R stimulation via the MAPK/ERK pathway, highlighting a mechanism by which the thymic microenvironment supports TEC survival [206]. Other essential TEC survival signals are provided through the activity of the linear ubiquitin assembly complex (LUBAC), which is composed of three proteins (HOIL‐1, HOIP, and Sharpin) and is required for activation of the NFκB pathway, among others [207]. TEC‐specific deficiency in HOIL‐1 caused severe thymic atrophy postnatally due to caspase‐8/MLKL‐dependent apoptosis/necroptosis of TEC. By contrast, loss of function in another LUBAC component, Sharpin, incurred a defect in the mTEC^low^ subset likely linked to impaired differentiation [207].

Although these findings highlighted key survival factors preventing TEC death, they do not reveal how TEC turnover is regulated in homeostasis. TEC‐specific ablation of essential regulators of apoptosis, autophagy, or necroptosis (an inflammatory form of receptor‐mediated cell death) demonstrated that only loss of BAX and BAK increased TEC number, specifically mTEC^low^ [208]. This finding indicates that the intrinsic pathway of apoptosis is a key regulator of steady‐state TEC turnover and is consistent with the hypothesis that much of the attrition of mTECs occurs in post‐AIRE cells. The extent to which apoptosis is required for TEC clearance and the role of efferocytosis in thymic function remain interesting outstanding questions, particularly in light of recent findings of an important role for dead cell clearance in regulating skin epithelial stem cell function [209].

Another important question is how cell death dynamics shape TEC responses to acute injury or thymic regeneration. The normally high rates of thymocyte apoptosis have been found to restrain a pro‐regenerative program that culminates in TEC activation [210]. Conversely, irradiation‐induced inflammatory death of thymocytes via pyroptosis has been reported to stimulate upregulation of FOXN1 by cTEC and regeneration via the purinergic receptor, P2Y2 [211]. However, whether changes in TEC survival modulate the acute injury response and thymic regeneration remains unknown. Acute insults certainly cause marked changes to the thymic microenvironment during the atrophy and regeneration phases [212]. Yet, in the context of the more gradual age‐related thymic involution, careful measurements of TEC found that morphological changes linked to metabolic damage, not alterations in cell number, were key aspects of atrophy and regeneration [91, 213]. These conclusions are consistent with the finding that thymic involution progresses normally in *Bax* ^Δ*Foxn1*^ *Bak* ^−/−^ despite the observed block in mTEC^low^ death [208]. Nevertheless, it remains possible that metabolic stress and oxidative damage within the aging thymus microenvironment can sensitize TECs to apoptosis upon acute insults [214]. Understanding how TEC death processes and clearance might impact thymic function could inform new therapeutic approaches for preserving or regenerating thymic function to restore T‐cell immunity.

## 12 Integration of Single Cell and Imaging Modalities to Resolve Thymic Function and Dysfunction

As outlined at the end of Section 4, a major technical limitation with single‐cell analysis of the thymic stroma is the poor recovery of cells and under‐representation of sensitive populations; an issue common to many other tissues. Major advances in 3D imaging, high parameter immunohistology, and spatial transcriptomics offer solutions to this problem and have driven recent discoveries into thymic stromal cell function.

Yayon et al. combined datasets derived from spatial transcriptomics, high parameter immunohistology, single‐cell RNAseq, and CITEseq analysis of fetal and pediatric human thymus [185]. By mapping these single‐cell data onto a common coordinate framework of the thymus, termed the cortico‐medullary axis, the authors could track inferred T‐cell differentiation trajectories and features of the stromal cues that guide them. Parenthetically, this concept for mapping thymic structure based on landmarks extends upon a prior innovative study quantifying thymic organization and microenvironmental defects [215]. The multimodal spatial approach mapped to the cortico‐medullary axis localized putative mcTEC progenitor cells, characterized the deep medullary distribution of mimetic cells, inferred differentiation trajectories of mTEC, and identified the distinct cellular neighborhoods established around Hassall's corpuscles [185]. In addition, recent advances in spatial proteomics using CODEX, single‐cell transcriptomic‐proteomic profiling (CITEseq), and chromatin accessibility analysis by ATAC‐seq highlighted sex‐based differences in the postnatal human thymus and identified specialized negative selection niches in proximity to Hassall's corpuscles [216]. Building on these impressive resources with analyses of thymi from adults and patients with defects in T‐cell differentiation will hopefully provide new discoveries into the genesis of thymic involution and dysfunction.

In our recent study, the application of new 3D imaging approaches has also revealed aspects of thymus biology that had been overlooked in prior histological and flow cytometric analyses. Lightsheet imaging of whole thymic lobes from a TEC reporter strain expressing a nuclear‐localized GFP revealed the striking accumulation of “high density” TEC regions in the involuting thymus [90]. These unusual structures were formed by dense conglomerates of TEC and excluded differentiating thymocytes. We found, by quantification, that these structures accumulated adjacent to medullary regions in the thymus of mice from 6 months and composed a substantial volume of the thymus by 1 year of age. Single‐cell RNAseq analysis of thymic stroma from aged mice revealed the emergence of two populations of transcriptionally distinct TEC unique to the involuting thymus, termed age‐associated TEC (aaTEC) [90]. Gene expression analysis revealed that these populations had lost critical TEC functions, such as antigen presentation, with aaTEC‐2 undergoing partial epithelial‐to‐mesenchymal transition and loss of EpCAM expression. Spatial transcriptomics and phenotypic analysis confirmed that aaTECs form the high‐density TEC regions resolved by 3D imaging, identifying a novel microenvironment of the involuted thymus [90].

**FIGURE 2:**
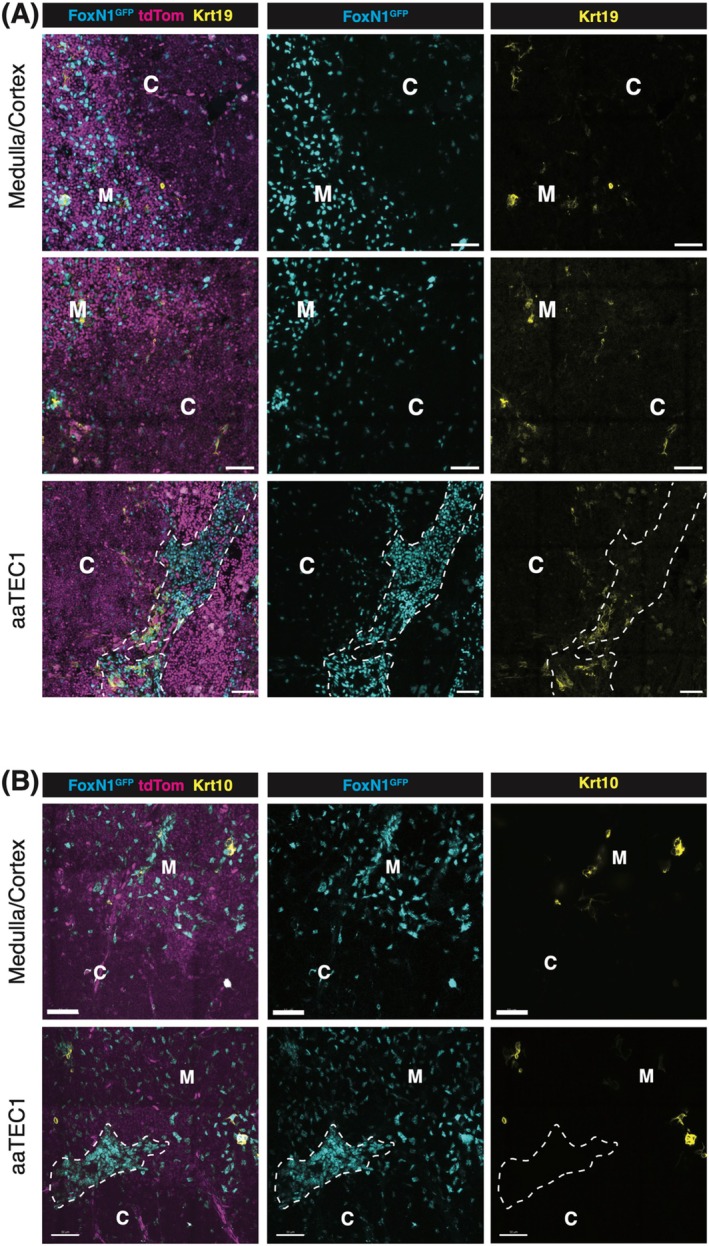
Representative confocal images of Krt19 (A) and Krt10 (B) expression in the thymus of aged Foxn1^nTnG^ mice. The medullary region is identified by high tdTomato expression and relatively high TEC cell density, whereas the cortical region is characterized by low tdTomato expression and lower TEC density. The aaTEC1 region is distinguished by a very high density of TECs, with the dotted line marking its boundary. C: Cortex; M: Medulla; Scale bar: 50 μm. Thymic lobes were fixed in paraformaldehyde (PFA) and sectioned into 200 μm slices using a vibratome. The sections were blocked with 0.3% Triton X‐100 in PBS and subsequently stained with specific antibodies diluted in 0.1% Triton X‐100 in PBS. Following staining, the sections were cleared transparent using EasyIndex optical clearing solution (LifeCanvas). Imaging was performed using a Zeiss LSM 980 confocal microscope, with representative images presented as 10 μm projections.

**FIGURE 3:**
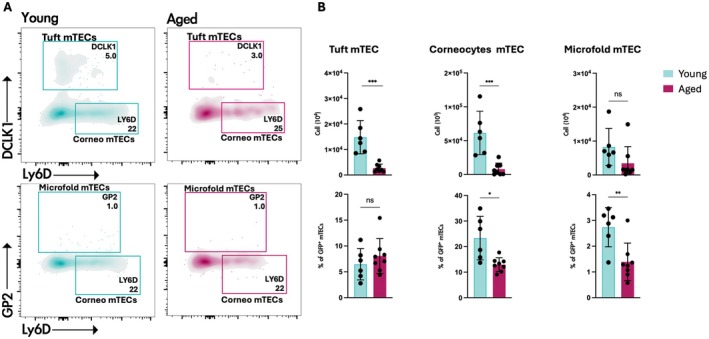
Reduced number of mimetic cells in young and aged *Foxn1* ^nTnG^ mice. (A) Representative contour plots identifying tuft, corneocyte, and microfold mimetic cells in thymi from 2‐month‐old or 18‐month‐old *Foxn1* ^nTnG^ mice. Cells were gated on CD45^−^/EpCAM^+^/GFP^+^ cells. (B) Quantification of mimetic cell subsets, presented as the total number of cells (upper panels) and the proportion of GFP^+^ mTECs (lower panels). Data are from 2‐month‐old (*n* = 6) and 18‐month‐old (*n* = 8) Foxn1^nTnG^ mice. Statistics were generated with two‐tailed paired *t* test. ns = non‐significant, *p = 0.03, **p = 0.002, ***p = 0.0002.

What is the origin and nature of aaTECs? RNA trajectory analyses imply that aaTEC may be the product of an alternative differentiation pathway derived from TEC progenitors/mTEC1 cells [90]. Alternatively, their expression of the tight junction component, Claudin‐3 [90], raises the possibility that aaTEC represents the accumulation of an arrested mTEC progenitor. Analysis of the expression of keratin‐10 (associated with post‐AIRE mTEC and corneocyte mimetic cells) or keratin‐19 (an mTEC‐biased progenitor in the embryo) demonstrated that neither wholly co‐localizes with aaTEC in the involuted thymus (Figure 2). Further studies will be required to track the origin of aaTEC1 and aaTEC2. Although these populations are clearly transcriptionally distinct from the characterized mimetic populations, questions remain about their potential relationships. In contrast to the expansion of aaTECs observed with thymic involution, we find that certain thymic mimetic cell populations decrease in number and proportion of total TEC with involution (Figure 3). This correlation may be consistent with a scenario where mTEC differentiation is skewed away from the conventional route terminating in mimetic cells toward an alternative aaTEC fate. Acute thymic damage induced by irradiation increased the number of aaTECs and the volume of this microenvironment, with evidence that these populations could co‐opt key growth factors from conventional TEC populations and slow thymic regeneration [90]. Overall, these findings raise questions about the phenotype, function, and origin of aaTECs and suggest that the process of thymic involution may, at least in part, be linked to an “in‐built” mechanism derived from alternative TEC differentiation.

## 13 Concluding Remarks

As in other fields, two key forces have propelled discoveries in thymic stromal biology: technology and argument. Data‐driven approaches benefit greatly from conceptual frameworks, such as the “progressive restriction” and “terminal differentiation” hypotheses that guided discoveries into AIRE's function and mimetic cells, or the various propositions offered to explain TEC progenitor function. These constructs challenge the field to apply new technologies to test, discard, and refine models of thymic function to create a synthesis that explains the many curious features of this organ. Numerous gaps remain in our knowledge that promise to keep the field busy for decades to come. How does positive selection work? Why does the thymus involute? Why is it so sensitive to damage? By what mechanisms does it regenerate? How does the stroma regulate T‐cell progenitor influx? How do variations in thymic function impact upon human susceptibility to autoimmune disease, and how do we measure these? Hypotheses will remain essential to addressing these and other problems, to forge further remarkable discoveries, and pave the way to modulating thymic function for clinical benefit.

## Conflicts of Interest

The Walter and Eliza Hall Institute of Medical Research receives milestone and royalty payments related to venetoclax. Employees are entitled to receive benefits related to these payments; D.H.D.G. reports receiving benefits. D.H.D.G. has received research funding from Servier.

## Data Availability

Data available on request from the authors.
